# The clinical learning environment, supervision and nurse teacher scale (CLES+T): psychometric properties measured in the context of postgraduate nursing education

**DOI:** 10.1186/s12912-020-00455-5

**Published:** 2020-07-06

**Authors:** Dorota Ozga, Aleksandra Gutysz-Wojnicka, Bogumił Lewandowski, Beata Dobrowolska

**Affiliations:** 1 Department of Emergency Medicine, Faculty of Medicine, University of Rzeszów, Pigonia 6 Street, 35-310 Rzeszów, Poland; 2 Department of Nursing, Faculty of Health Sciences, Collegium Medicum, University of Warmia and Mazury in Olsztyn, Zolnierska 14c Street, 10-561 Olsztyn, Poland; 3 Department of Development in Nursing, Faculty of Health Sciences, Medical University of Lublin, Staszica Street 4-6, 20-081 Lublin, Poland

**Keywords:** Postgraduate nursing education, Clinical learning environment, Psychometric properties, Polish version of CLES+T

## Abstract

**Background:**

The Clinical Learning Environment, Supervision and Nurse Teacher Scale **(**CLES+T) instrument is internationally used for the evaluation of clinical learning environment in undergraduate nursing education. However, no evidence is available on the possible applications of this instrument in the context of postgraduate nursing education.

**Purpose:**

To examine the basic psychometric properties of CLES+T in the context of clinical postgraduate nursing education in Poland.

**Methods:**

Study among a sample of 417 nurses participating in the clinical postgraduate training in Poland.

**Results:**

Cronbach’s alpha for the total scale was 0.97. A five-factor structure was confirmed in accordance with the assumptions adopted by the authors of the original version of the scale. Cronbach’s alpha coefficient for the Polish version of the CLES+T subscales ranged from 0.83 (*Nursing care on the ward*) to 0.95 (*The content of supervisory relationship*). The mean results for individual subscales ranged from 4.52 ± 0.63 for *nursing care on the ward* to 4.73 ± 0.45 for *role of the nurse teacher*.

**Conclusions:**

Having shown satisfactory psychometric properties, CLES+T can be considered a useful instrument to assess those elements of clinical learning environment which are important for the assurance of education quality at the postgraduate level.

## Background

A relatively strong emphasis is placed on the importance of clinical learning environment (CLE) in the provision of undergraduate nursing education [1–3]. This part of training, however, appears to have fundamental significance also in postgraduate nursing education. Developing advanced nursing roles, whether during master studies or various clinically based specialisation programs, requires high quality and effective CLE where trainees are able to advance their competences and increase confidence as independent nursing professionals [4, 5]. Therefore, any instruments which allow one to measure the quality of CLE in postgraduate settings and to identify those CLE elements which need to be addressed and improved are valued. Despite there being several globally available questionnaires for assessing nursing students’ perception of the clinical environment, these are generally used as part of undergraduate education [6]. One of them is the Clinical Learning Environment, Supervision and Nurse Teacher Scale (CLES+T) [7] accepted as a tool commonly used at the international level to evaluate CLE [8]. This scale includes items that enable students to assess five basic elements of clinical learning such as supervision and/or mentorship, role of the nurse teacher, a learning-conducive atmosphere on the ward, nursing care provided on the ward, and the leadership style of the ward manager [7]. Considering the fact that nursing clinical education should be organised in exemplary facilities, where nursing care is conducted according to accepted professional standards, the style of ward management promotes the quality of care and satisfaction of nursing staff, nurse-specialists willingly share their knowledge with nurse-trainees, and the clinical mentor and the nurse teacher are professionals actively involved in the clinical teaching process [4, 9] CLES+T is applicable to monitor postgraduate education provided in the form of clinical training. This helps to keep CLE at an acceptable level, impact nurses’ satisfaction from postgraduate nursing education and thus stimulate nurses to engage in different postgraduate courses in the future [10]. However, to our best knowledge, no study has been developed to utilise the CLES+T scale in the context of postgraduate nursing education. The aim of our research was, therefore, to examine the psychometric properties of the Polish version of the CLES+T instrument in postgraduate settings.

Participation in postgraduate nursing education is considered to be every nurse’s obligation, pursued in order to further one’s competences necessary to ensure the provision of high-quality nursing care in a more independent and confident manner [5, 11]. A broad variety of systems and forms of postgraduate education of nurses exist across the world and many new trends are observed as regards its organisation [12, 13]. It can be organised as university-based program, hospital-based specialisation program, or residency programs offered by healthcare institutions [9, 10]. Furthermore, expectations for promoting the advancement of nursing competences are growing, not only because of demographic challenges such as population ageing, but also due to shortages in healthcare professionals [13]. There is quite ample evidence for the multifaceted nature of benefits offered by nurses’ participation in postgraduate education: it encourages critical thinking, clinical reasoning, and advanced nursing skills; it contributes to patients’ satisfaction and health outcomes, and helps avoid medical errors; finally, it fosters nurses’ professional growth and sense of fulfilment [5, 10, 14–16].

The development of advanced nursing competences within postgraduate education is more effective if implemented in both theoretical and practical training conditions and if a goal-oriented mentoring of trainees is given a major role in the student’s overall professional development [3]. A considerable part of postgraduate nursing education in a variety of nursing disciplines takes place within clinical environment in the form of practical training [4, 9, 17]. However, there is not much research investigating teaching-learning process and mentoring patterns in the context of postgraduate education [4, 9]. As an example, considering the importance of the quality and governance of clinical learning in nurse practitioner education, Gardner and colleagues [4] are designing the study to provide a theoretically informed clinical educational model to support learning and teaching in advanced specialty postgraduate education. Nyhagen and Strøm [9], on the other hand, using the focus group method, investigated postgraduate students’ perception of one-to-one precepting during critical care education.

It is underlined that nurses need to be educated in CLE, which should help them develop their potential and become advanced practitioners [4, 5]. Here, the role of nurse educators, responsible for building positive learning environments, is highlighted [10]. In addition, considering nurses’ obligation to continue their professional development, their experience regarding CLE and mentoring models have an impact on their decisions and motivation as regards further education [10]. For this reason, CLE elements such as pedagogical atmosphere on the ward or other healthcare facilities, the leadership style of the ward manager, nursing care on the ward, supervisory and/or mentoring patterns, and the role of the nurse teacher, identified and specified by Saarikoski and colleagues [7] in the CLES+T scale, assume central importance and should be monitored with the same attentiveness as in the case of practical training during undergraduate nursing education. This is important also because participants of postgraduate courses are registered working nurses with various levels of professional experience and expectations. Therefore, their perspective is different from that of pre-registration student nurses.

There has been a variety of undergraduate nursing students’ cohorts researched with the use of CLES+T across cultures to evaluate CLE in different clinical settings showing the correlation between well assessed CLE with mentor and nurse teacher role, and nursing students’ satisfaction and outcomes in clinical training [3, 18, 19]. Also complementariness of the CLES+T scale and the Cultural and Linguistic Diversity Scale (CALDs) designed to be used together to assess CLE by the international nursing students was studied [20]. Additionally, all current validation studies of CLES+T resulted in good psychometric properties, providing an opportunity for an effective assessment of CLE in various cultural environments [21–24].

In Poland, nurses can participate in four nationally regulated forms of postgraduate education, the same for individuals with BSc and Master’s degrees [17, 25]: *specialist training in nursing* (2 years), *qualifying courses* (6 months), *specialised courses* (minimum 4 weeks), and *additional training courses* (short forms of training). Each of these courses provides nurses with a different range of clinical competences and independence in their professional work with patients [26]. The majority of training hours are delivered in clinical settings, e.g. practical training at specialised centres under the supervision of experienced supervisors and teachers, with a view to fostering the acquisition of specialist, advanced nursing competences in a variety of nursing disciplines [27]. This education is usually provided by accredited centres for nurses and midwives’ postgraduate education, often university based. Compared to Polish system, in the other systems developed internationally the acquisition of advanced clinical competences by nurses is often organised at the Master’s level and concludes with the qualification of Advanced Practice Nurse (APN) across various nursing disciplines [9, 28].

### Aim

The aim of the study was to examine the psychometric properties of the Polish version of the Clinical Learning Environment, Supervision and Nurse Teacher Scale (CLES+T) in the context of postgraduate nursing education.

## Methods

### Design

A cross-sectional observational study among a convenience sample of nurses participating in postgraduate training in the region of South Poland. The study was reported in accordance with the STrengthening the Reporting of OBservational studies in Epidemiology (STROBE) checklist [29].

### Participants and setting

460 participants of a specialised course on *Cardiopulmonary resuscitation for nurses and midwives* were contacted and invited to participate in the survey. 420 questionnaires were returned (response rate of 91.30%) and, having been checked for completeness, 417 qualified for the final analysis. The 1:10 rule [30] was adopted, requiring that the number of respondents be at least ten times higher than the number of the variables (items on the scale). Therefore, a sample of 460 nurses participating in the study reached the minimum criterion.

The study evaluated the practical part of the training (clinical internship) lasting 30 h, during a specialised course on cardiopulmonary resuscitation for nurses and midwives, the content of which was approved by the Minister of Health on August 31, 2017 [31]. The place of the internship was an intensive care unit (ICU) in one of hospitals in South Poland. The internship was carried out as part of the Second Teaching Module: Advanced Resuscitation Practices – ALS. Trainees were mentored by one mentor – employed by the hospital where the internship took place, and it was organised in the form of group mentoring with a ratio of 1:5. Completing the course on *Cardiopulmonary resuscitation* by nurses and midwives is recommended by employers and it is also obligatory part of specialist training in nursing/midwifery. In both cases, it is organised by registered centres for postgraduate education for nurses and midwives, and it is based on a nationally agreed program [31].

### Research instrument

The Clinical Learning Environment, Supervision and Teacher Scale (CLES+T) was developed in 2000/2002 following thorough theoretical considerations. Since then it has evolved and has been refined [7, 32]. The original version of the scale, CLES, served as the basis for the creation of CLES+T, now available in multiple languages [33]. In this study, we used the CLES+T version from 2008 [7]. The current version of the scale consists of 34 items with five sub-dimensions: *pedagogical atmosphere* (9 items), *leadership style of the ward manager* (4 items), *nursing care on the ward* (4 items), *the content of supervisory relationship* (8 items), and *role of the nurse teacher* (9 items) [7]. All items are rated based on a five-point Likert scale (1 = fully disagree, 2 = disagree to some extent, 3 = neither agree nor disagree, 4 = agree to some extent, and 5 = fully agree) [7].

### Content validity of an instrument

After obtaining permission from the author of the CLES+T scale, the procedure of translation from its original, English version was commenced. The instrument was translated into Polish independently by two professional linguists. The two translations were then compared, and on this basis one coherent version was agreed on and translated back into English. Any additional doubts regarding differences in terms and phrases were discussed within a group of three researchers – the authors of this study. Eventually, the final version of the Polish CLES+T instrument was accepted.

Since our study pertains to postgraduate nursing education, in the Polish version of the CLES+T used in this environment we have added some terms to clarify the settings to which this scale is referring. These in particular include the sub-dimensions *the content of supervisory relationship* and *role of the nurse teacher,* in which the titles of supervisor and nurse teacher were clarified to match the Polish conditions: (1) *an internship supervisor* (clinical mentor) – a person employed by a healthcare provider to supervise and evaluate students during their clinical training in hospital or primary healthcare settings; and (2) *a postgraduate course coordinator* (nurse teacher) – a person employed by a postgraduate education institution responsible for organising both theoretical and practical training; co-decides about the selection of teaching staff, evaluates practical training institutions according to the specifics and organisation of classes, assists in solving problems, and provides individual consultations to the participants of the course [31].

### Data collection

One of the researchers approached students during their final classes in simulated conditions at the postgraduate education centre for nurses and midwives. Considering the double role of the researcher who was at the same time a teacher and in order to avoid any pressure and discomfort for the students invited to participate in the study, having been explained in detail the goals of the study and the protocol, the students received questionnaires with blank envelopes and were asked to complete these questionnaires and deliver them within one week to the centre’s administration office. The questionnaires were being distributed from January 2016 to September 2017.

### Ethical considerations

Participants were invited to take part in the study on a voluntary basis. All of them were informed about the aim of the study and the research protocol. Participants were assured that data collected during the study was anonymous and would be protected in terms of privacy. They were also notified of their right to withdraw from the study at any time. Consent to participate in the study was given verbally by the students, who had to submit the questionnaires in anonymous envelopes to the administration office of the postgraduate education centre for nurses and midwives, in accordance with instructions provided by the teacher. To avoid any pressure, no reminders were sent to the students, except for the information given at the first meeting. The researcher who collected data was not involved in the practical training of the students in clinical environment but was only responsible for the laboratory/theoretical part of one module of the course. The research protocol, together with the respondent consent form, were approved by the Ethical Commission at the University of Rzeszów (Resolution No 6/9/2017). Following data collection, the material was entered into an Excel spreadsheet and stored on a password-protected computer at the first author’s university. Under the university’s policy, data will be archived for 30 years, both in paper and electronic versions (recorded on a CD).

### Data analysis

An exploratory factor analysis based on a principal component analysis (PCA), followed by Promax rotation (with Kaiser normalisation) were performed to confirm the adequate number of factors in the data. It was accepted that eigenvalues should be greater than 1.0 and the cut-off value for displaying factor loadings was set to 0.3. To assess the validity of the scale, the Cattell’s scree test was also applied. The discriminatory power of positions was measured. Bartlett’s Test of Sphericity between the items and Kayser-Meyer-Olkin test (KMO) for sampling adequacy were used. Cronbach’s alpha was applied to assess the reliability and internal consistency of the Polish version of CLES+T. Values greater than 0.7 were considered acceptable. Additionally, a descriptive statistics analysis was performed*.* Statistical analyses were carried out using PS IMAGO.

## Results

### Participants and clinical placement

The research group consisted of 417 nurses from South Poland – women (98.80%, *n* = 412) and men (1.20%, *n* = 5). The youngest of the respondents was 23 years old and the oldest 59 years old, with a mean age of 36.7 years ±10.3. The mean work experience as a nurse was 12.9 years ±11.3.

The internship took place at one of the largest hospitals (891 hospital beds) in Subcarpathian Voivodship, South Poland. Students underwent clinical training at the Anesthesiology and Intensive Care Unit with the Center for Acute Poisonings (25 hospital beds).

### Principal component analysis

Sample adequacy was confirmed by means of the KMO test (0.96) and Bartlett’s test of sphericity (*χ*^2^ = 11,890.304, Df = 561, *p* < .001), indicating that correlations between items were large enough. These findings revealed the absence of correlations between variables and the factor analysis could be performed. Table 1 shows the results of the exploratory factor analysis. The five factors generated explained 67.9% of the variance and all eigenvalues > 1. One item worded “The WM regarded the staff on her/his ward as a key resource” from the component “leadership style of the ward manager” loaded on “the content of supervisory relationship” as well (Table 1).

**Table 1:** Factor loadings and inter-factor correlation coefficient

Items of CLES+T	Factor 1	Factor 2	Factor 3	Factor 4	Factor 5
The content of supervisory relationship					
My supervisor showed a positive attitude towards supervision	**0.741**	0.153	0.062	−0.113	− 0.021
I felt that I received individual supervision	**0.842**	−0.019	0.075	−0.042	−0.064
I continuously received feedback from my supervisor	**0.881**	−0.069	0.109	−0.078	0.034
Overall I am satisfied with the supervision I received	**0.891**	−0.094	0.096	0.045	−0.083
The supervision was based on a relationship of equality and promoted my learning	**0.833**	−0.003	0.045	0.062	0.030
There was a mutual interaction in the supervisory relationship	**0.772**	0.102	−0.141	0.067	0.107
Mutual respect and approval prevailed in the supervisory relationship	**0.766**	0.187	−0.145	0.121	−0.048
The supervisory relationship was characterized by a sense of trust	**0.739**	0.128	− 0.108	0.042	0.033
Role of the nurse teacher					
In my opinion, the nurse teacher was capable of integrating theoretical knowledge and everyday practice of nursing	−0.083	**0.905**	−0.096	0.044	0.024
The teacher was capable of operationalizing the learning goals of this clinical placement	0.051	**0.550**	−0.017	0.230	0.012
The nurse teacher helped me to reduce the theory-practice gap	0.068	**0.622**	0.007	0.166	0.066
The nurse teacher was like a member of the nursing team	0.224	**0.456**	0.039	0.105	0.091
The nurse teacher was capable of giving his or her pedagogical expertise to the clinical team	0.284	**0.337**	−0.035	0.222	0.068
The nurse teacher and the clinical team worked together in supporting my learning	0.261	**0.510**	−0.016	0.075	0.114
The common meetings between myself, my mentor, and my nurse teacher were comfortable experiences	0.094	**0.846**	0.052	−0.132	− 0.050
In our common meetings I felt that we were colleagues	0.125	**0.661**	0.274	−0.266	0.044
The focus of the meetings was on my learning needs	0.021	**0.666**	0.331	−0.113	−0.010
Pedagogical atmosphere					
The staff members were easy to approach	−0.076	0.342	**0.780**	−0.093	−0.252
I felt comfortable going to the ward at the start of my shift	0.053	−0.129	**0.669**	−0.294	0.529
During staff meetings (e.g., before shifts) I felt comfortable taking part in the discussions	0.105	−0.126	**0.778**	−0.075	0.222
There was a positive atmosphere on the ward	−0.044	0.178	**0.611**	0.056	0.145
The staff members were generally interested in student supervision	0.007	0.086	**0.466**	0.284	0.025
The staff learned to know the students by their personal names	0.028	−0.326	**0.318**	0.234	0.364
There were sufficient meaningful learning situations on the ward	−0.258	0.153	**0.522**	0.475	0.014
The learning situations were multidimensional in terms of content	0.098	−0.017	**0.517**	0.305	0.068
The ward can be regarded as a good learning environment	0.114	−0.013	**0.673**	0.331	−0.295
Nursing care on the ward					
The ward’s nursing philosophy was clearly defined	0.152	0.010	0.022	**0.508**	0.209
Patients received individual nursing care	−0.040	−0.012	−0.081	**0.782**	0.087
There were no problems in the information flow related to patients’ care	0.111	0.015	0.032	**0.820**	−0.103
Documentation of nursing (e.g., nursing plans, daily recording of nursing procedures, etc.) was clear	0.227	−0.200	0.172	**0.630**	−0.035
Leadership style of the ward manager					
The WM regarded the staff on her/his ward as a key resource	−0.215	0.158	−0.024	**0.466**	**0.382**
The WM was a team member	−0.012	0.065	−0.121	0.036	**0.943**
Feedback from the WM could easily be considered as a learning situation	0.005	0.115	−0.015	0.004	**0.860**
The effort of individual employees was appreciated	0.047	0.087	0.079	0.171	**0.593**
Eigenvalues	**17.37**	**1.99**	**1.35**	**1.30**	**1.09**
Total percentage and cumulative addition	**51.1%**	**5.8%**	**4.0%**	**3.8%**	**3.2%**
	S-d1	S-d2	S-d3	S-d4	S-d5
S-d1	1.000				
S-d2	0.695	1.000			
S-d3	0.605	0.541	1.000		
S-d4	0.649	0.607	0.581	1.000	
S-d5	0.563	0.537	0.554	0.569	1.000
Cronbach’s alpha for each factor	0.946	0.925	0.893	0.834	0.883
Cronbach’s alpha on total scale	0.967				

The items of CLES+T scale ‘reprinted from International Journal of Nursing Studies, Vol. 45, Issue 8, Mikko Saarikoski, Hannu Isoaho, Tony Warne, Helena Leino-Kilpi, The nurse teacher in clinical practice: developing the new sub-dimension to the clinical learning environment and supervision (CLES) scale, pp. 1235–1236, Copyright (2020), with permission from Elsevier’

Cronbach’s alpha coefficient for the Polish version of the CLES+T sub-dimensions ranged from 0.83 (*Nursing care on the ward*) to 0.95 (*The content of supervisory relationship*). (Table 1).

The item means varied between 4.24 and 4.79 (on a scale of 1–5). Means for each sub-dimension were also calculated. The highest mean was calculated for the sub-dimension *role of the nurse teacher*: 4.73 ± 0.45, then for *the content of supervisory relationship*: 4.70 ± 0.53, and for *leadership style of the ward manager*: 4.58 ± 0.65. The lowest means were calculated for the sub-dimensions *pedagogical atmosphere*: 4.56 ± 0.54, and *nursing care on the ward*: 4.52 ± 0.63 (Table 2).

**Table 2:** Means calculated for the CLES + T items and sub-dimensions

Items	M	SD	Min	Max	Q1	Me	Q3
*Pedagogical atmosphere*							
Item 1	4.74	0.61	1.00	5.00	5.00	5.00	5.00
Item 2	4.58	0.77	1.00	5.00	4.00	5.00	5.00
Item 3	4.36	0.83	1.00	5.00	4.00	5.00	5.00
Item 4	4.66	0.69	2.00	5.00	5.00	5.00	5.00
Item 5	4.61	0.66	1.00	5.00	4.00	5.00	5.00
Item 6	4.24	1.09	1.00	5.00	4.00	5.00	5.00
Item 7	4.61	0.61	2.00	5.00	4.00	5.00	5.00
Item 8	4.58	0.66	2.00	5.00	4.00	5.00	5.00
Item 9	4.68	0.56	2.00	5.00	4.00	5.00	5.00
Pedagogical atmosphere	**4.56**	**0.54**	**2.11**	**5.00**	**4.33**	**4.78**	**5.00**
*Leadership style of the ward manager*							
Item 1	4.64	0.63	1.00	5.00	4.00	5.00	5.00
Item 2	4.54	0.82	1.00	5.00	4.00	5.00	5.00
Item 3	4.59	0.78	1.00	5.00	4.00	5.00	5.00
Item 4	4.56	0.79	1.00	5.00	4.00	5.00	5.00
Leadership style of the ward manager	**4.58**	**0.65**	**1.50**	**5.00**	**4.25**	**5.00**	**5.00**
*Nursing care on the ward*							
Item 1	4.54	0.73	1.00	5.00	4.00	5.00	5.00
Item 2	4.38	0.93	1.00	5.00	4.00	5.00	5.00
Item 3	4.54	0.72	1.00	5.00	4.00	5.00	5.00
Item 4	4.61	0.69	2.00	5.00	4.00	5.00	5.00
Nursing care on the ward	**4.52**	**0.63**	**1.50**	**5.00**	**4.25**	**4.75**	**5.00**
*The content of supervisory relationship*							
Item 1	4.72	0.59	1.00	5.00	5.00	5.00	5.00
Item 2	4.62	0.64	1.00	5.00	4.00	5.00	5.00
Item 3	4.66	0.64	1.00	5.00	4.00	5.00	5.00
Item 4	4.69	0.63	1.00	5.00	5.00	5.00	5.00
Item 5	4.68	0.66	1.00	5.00	5.00	5.00	5.00
Item 6	4.72	0.63	1.00	5.00	5.00	5.00	5.00
Item 7	4.76	0.58	1.00	5.00	5.00	5.00	5.00
Item 8	4.77	0.56	1.00	5.00	5.00	5.00	5.00
The content of supervisory relationship	**4.70**	**0.53**	**1.00**	**5.00**	**4.63**	**5.00**	**5.00**
*Role of the nurse teacher*							
Item 1	4.77	0.52	2.00	5.00	5.00	5.00	5.00
Item 2	4.73	0.59	2.00	5.00	5.00	5.00	5.00
Item 3	4.73	0.57	2.00	5.00	5.00	5.00	5.00
Item 4	4.71	0.57	2.00	5.00	5.00	5.00	5.00
Item 5	4.70	0.58	2.00	5.00	5.00	5.00	5.00
Item 6	4.72	0.60	1.00	5.00	5.00	5.00	5.00
Item 7	4.79	0.50	2.00	5.00	5.00	5.00	5.00
Item 8	4.66	0.67	1.00	5.00	4.00	5.00	5.00
Item 9	4.79	0.53	2.00	5.00	5.00	5.00	5.00
Role of the nurse teacher	**4.73**	**0.45**	**2.00**	**5.00**	**4.67**	**4.89**	**5.00**

*M-mean; SD-standard deviation; Min – minimum; Max – maximum; Me- median; Q1 - lower quartile; Q3 - upper quartile*

## Discussion

Having been translated into more than 27 languages, CLES+T is now used in over 40 countries [22]. In this study we propose the Polish version of this research instrument, as it displays good validity and reliability levels and can be recommended for use in the evaluation of CLE, supervision, and the role of nurse teacher in postgraduate nursing education. Cronbach’s alpha for the Polish version of the scale was 0.97. Cronbach’s alpha coefficient for the CLES+T sub-dimensions ranged from 0.83 to 0.95. These results are in line with other validation studies performed in culturally similar environments. For example, in the Slovak version, Cronbach’s alpha of the 34-item CLES+T was 0.94, and Cronbach’s alphas for each of the five subscales ranged from 0.80 to 0.97 [21]. In the Croatian version, the overall Cronbach’s alpha of 33 items was 0.97, and Cronbach’s alphas for each of the subscales ranged from 0.77 to 0.96 [1]. In the Slovenian version, the overall Cronbach’s alpha of 34 items was 0.96, and Cronbach’s alphas for the subscales ranged from 0.78 to 0.94 [22]. However, it should be underlined that all of the previous studies took place in an undergraduate education context.

In our own research, a five-factor structure was confirmed in accordance with the assumptions adopted by the authors of the original version of the scale. The five-factor model with the explanation of 67.9% of the total variance in our study was comparable to the factorial models of other studies in Europe. To provide some examples – in the original, Finnish version: 64% of the total variance [7], in the Spanish version: 66.4% of the total variance [34], in the Cypriot version: 67.4% of the total variance [35], and in the Slovenian version: 67.69% of the total variance [22]. The four-factor model of CLES+T resulted from Croatian validation study and explained 71.5% of the total variance [1], in Austrian studies with explanation of 73% of the total variance [2], and in German studies with explanation of 72.85% of the total variance [36].

The strongest factor in studies on the Polish version of CLES+T was the sub-dimension *the content of supervisory relationship* with high loading from 0.741 to 0.891, explaining 51.1% of variance. The sub-dimension *role of the nurse teacher* was found to be the second strongest factor in our study, with loading from 0.337 to 0.905 and the explanation of 5.8% of variance. This is in line with the Cypriot study in which this factor explained 11.02% of variance [35].

Considering these two factors as being the strongest in our study, we can assume that both, the *internship supervisor* (clinical mentor) and *course coordinator* (nurse teacher), are essential in successful clinical postgraduate education. Their high position is related to the scope of their responsibility. In the case of internship supervisor, it is mentoring as well as evaluating students’ achievements in clinical setting, while in the case of course coordinator, it is organising the whole training; selecting teaching staff, evaluating practical training institutions, being available and assisting in solving problems of postgraduate students [31]. The mentorship approach and the role of nurse teacher are acknowledged in undergraduate nursing education [19, 37], and this is visible in other validation studies of CLES+T in which these factors were also strong [7, 34–36, 38, 39].

The weakest factor in this study was the *leadership style of the ward manager* (3.2% of variance explained), a result similar to that in the Cypriot studies [35] and opposite to the German studies in which this factor was the second strongest [36]. In other studies, the weakest factor was usually *nursing care on the ward* [7, 34, 39]. Considering the weak results of *the leadership style of the ward manager* factor in our study, the possible explanation can lie in the working experience of postgraduate study participants as registered nurses (in our case average work experience of nurses was nearly 13 years) and thus their realistic evaluation of management style of ward manager and also his/her role in postgraduate clinical education. In undergraduate nursing education, Pitkänen and colleagues [3] indicated that students nurses assessed lower the management style of the ward manager than students from other healthcare programs, which may show that future nurses recognise the role of ward manager in creating educational atmosphere and assess it in a more reflective way.

The individual item means in our study varied between 4.24 and 4.79 and can be considered as relatively high, especially when compared with Swedish results where the individual item means ranged from 2.4 to 4.4 [38]. The mean results for individual subscales in Polish research were also calculated, as follows: for *pedagogical atmosphere*: 4.56 ± 0.54, for *leadership style of the ward manager*: 4.58 ± 0.65, for *nursing care on the ward*: 4.52 ± 0.63 (the lowest), for *the content of supervisory relationship*: 4.70 ± 0.53, and for *role of the nurse teacher*: 4.73 ± 0.45 (the highest). These results show that the work of educators responsible for the entire clinical training process (an internship supervisor and a postgraduate course coordinator) is duly recognised and acknowledged. In a Spanish study among nursing students, the means for all subscales were generally lower than in our study. The lowest mean was calculated also for the subscale *nursing care on the ward*: 3.38 ± 1.01, while the highest for *pedagogical atmosphere*: 4.15 ± 0.63 [34]. At the same time, in a Finnish study involving 1973 healthcare students, similarly to our results, the means for subscales were relatively high, although still lower than in the Polish one. As opposed to our study, however, the highest mean was calculated for *nursing care on the ward*: 4.56 ± 0.55 and the lowest for *role of the nurse teacher*: 3.98 ± 0.87 [3]. It is difficult to explain the high results in our study for each subscale of the CLES+T. This is specifically interesting, because our respondents were mature students with previous educational experience, who, presumably, have certain expectations regarding the organisation and course of training. What is important and may contribute to a high rating, especially for the subscales pertaining to supervisory relationship and the role of nurse teacher, is that every centre for postgraduate education of nurses and midwives is obliged by law to have quality assurance systems in place. Therefore, every *internship supervisor* (clinical mentor) and *postgraduate course coordinator* (nurse teacher) are regularly assessed by students and, depending, on the results of such assessments, the centre decides whether to continue cooperation with them or not [25].

### Limitations

This study has its limitations, the main one being the convenience sampling method. Nurses taking part in the study were recruited from only one region of Poland and one educational institution which, in the case of our study, was a postgraduate education centre for nurses and midwives. Therefore, the sample of nurses was too homogenous to allow the formulation of conclusions of a more general nature. Research in a more diverse group of respondents is recommended. Additionally, the test-retest reliability of the scale – an important measure for the scale development – was not assessed in our study and it is recommended for future research.

## Conclusions

The Polish version of CLES+T shows satisfactory psychometric properties. Having a validated CLES+T scale creates an opportunity for postgraduate nursing education providers, as they can use it to monitor and evaluate elements of CLE and mentoring patterns, which helps to improve those aspects which revealed to be weak. It is also an opportunity for researchers, as it enables conducting national and international comparative studies regarding the quality of CLE at the postgraduate level.

Results of this study support mentorship approach in the nursing education at the postgraduate level and the role of postgraduate course coordinator (nurse teacher). This is a strong signal for organisers of postgraduate nursing education to further invest in this aspect of clinical training.

Our study findings indicate that despite the systems of postgraduate nursing education internationally are different, the clinical training is its vital part, therefore the CLE, supervisory/mentoring and nurse teacher role may be evaluated using CLES+T.

## Data Availability

All data generated or analysed during this study available from the corresponding author on reasonable request.

## Abbreviations

CLES+T: The Clinical Learning Environment, Supervision and Nurse Teacher Scale
CLE: Clinical Learning Environment
CALDs: The Cultural and Linguistic Diversity Scale
APN: Advanced Practice Nurse
STROBE: The STrengthening the Reporting of OBservational studies in Epidemiology
ICU: Intensive care unit
ALS: Advanced Resuscitation Practices
PCA: Principal component analysis
KMO: Kayser-Meyer-Olkin test
